# Lifetime prevalence and potential determinants of psychotic experiences in the general population of Qatar

**DOI:** 10.1017/S0033291719000977

**Published:** 2020-05

**Authors:** Salma M. Khaled, Stacy Schantz Wilkins, Peter Woodruff

**Affiliations:** 1 Social and Economic Survey Research Institute, Qatar University, Doha, Qatar; 2 Greater Los Angeles Veterans Affairs Medical Center, Los Angeles, CA, USA; 3 Department of Medicine, David Geffen School of Medicine at University of California, Los Angeles, Los Angeles, CA, USA; 4 Department of Psychiatry, Hamad Medical Corporation, Doha, Qatar; 5 Weil Cornell – Medicine, Education City, Qatar Foundation, Doha, Qatar; 6 National Institute of Health Research – Sheffield Biomedical Research Centre, University of Sheffield, Sheffield, UK

**Keywords:** Arab, culture, ethnicity, migration, odd beliefs and magical thinking, prevalence, psychotic experiences

## Abstract

**Background:**

To estimate the lifetime prevalence and potential determinants of psychotic experience(s) (PEs) in the general population of Qatar – a small non-war afflicted, conservative, high-income, middle-eastern country with recent rapid urbanization including an influx of migrants.

**Methods:**

A probability-based sample (*n* = 1353) of non-migrants and migrants were interviewed face-to-face and administered a 7-item psychosis screener adapted from the Composite International Diagnostic Interview, the Kessler 6-item psychological distress scale, and the 5 items assessing odd (paranormal) beliefs and magical thinking (OBMT) from the Schizotypal Personality Questionnaire. Using bivariate and logistic regression analyses, lifetime prevalence rates of PEs were estimated then compared before and after adjustment for socio-demographics, Arab ethnicity, psychological distress, and OBMT.

**Results:**

Prevalence of PEs was 27.9%. Visual hallucinations were most common (12.8%), followed by persecutory delusions (6.7%) and auditory hallucinations (6.9%). Ideas of reference (3.6%) were least prevalent. PEs were significantly higher in Arabs (34.7%) compared with non-Arabs (16.4%, *p* < 0.001) with the exception of ideas of reference and paranoid delusions. Female gender was associated with a higher prevalence of PEs in the Arab group only (*p* < 0.001). Prevalence of PEs was significantly higher among Arabs (48.8% *v.* 15.8%, *p* < 0.001) and non-Arabs (35.2% *v.* 7.3%, *p* < 0.001) with OBMT. Arab ethnicity (OR = 2.10, *p* = 0.015), psychological distress (OR = 2.29 *p* = 0.003), and OBMT (OR = 6.25, *p* < 0.001) were independently associated with PEs after adjustment for all variables.

**Conclusions:**

Ethnicity, but not migration was independently associated with PEs. Evidence linking Arab ethnicity, female gender, and psychological distress to PEs through associations with OBMT was identified for future prospective investigations.

## Introduction

Psychotic experience(s) (PEs) are delusions and hallucinations that occur along a continuum within the general population (van Os *et al*., 2008; Verdoux and van Os, 2002). Schneider was the first to introduce the concept of ‘first-rank’ symptoms of schizophrenia that included psychotic phenomena (Schneider, 1959). These also commonly occur in healthy populations (DeVylder *et al*., 2016; Rössler *et al*., 2007; Yung and Lin, 2016). However, studies show that individuals with PEs are at increased risk of psychotic disorder, including schizophrenia (Fusar-Poli *et al*., 2012; Kaymaz *et al*., 2012). Significant predictors of whether an individual with PEs develops a psychotic disorder include: frequency, intensity and persistence of PEs, related psychological distress, schizotypal personality traits (including odd beliefs and magical ideation), cognitive dysfunction, exposure to traumatic events, and genetic risk (Freeman and Fowler, 2009; Kaymaz *et al*., 2012; McGrath *et al*., 2017; Raine, 1991; van Os *et al*., 2010, 2017; Wigman *et al*., 2012; Yung *et al*., 2009; Yung and Lin, 2016).

PEs are prevalent globally with population estimates varying widely depending on the assessment instruments used and underlying culture with a median prevalence range of 5.0% to 7.4% and a median incidence rate range of 2.5% to 3.0% (Linscott and van Os, 2013; van Os *et al*., 2008). Brief screening instruments find a higher prevalence of PEs than formal diagnostic assessment (Kessler *et al*., 2005). Studies on the prevalence of at least one psychotic symptom worldwide range from 0.8% to 45.8% (DeVylder *et al*., 2016; Larøi *et al*., 2014; Nuevo *et al*., 2012). The World Mental Health (WMH) Surveys found a mean lifetime prevalence of ever having a PE across 18 countries globally of 5.8%, hallucinations (5.2%) being more common than delusions (1.3%) (McGrath *et al*., 2015). Lifetime PE prevalence is higher in middle and high-income countries, women, non-married, not employed, and those with low family income (McGrath *et al*., 2015).

PEs and psychotic disorders have also been found to be higher in many migrants and minority migrant populations, with elevated risk occurring in the first and second generation of migrant populations (Morgan *et al*., 2010). A diverse range of social factors have been implicated in elevated rates of PEs in migrants including small ethnic density, stress, perceived discrimination, and cumulative negative social experience (DeVylder *et al*., 2016; Leaune *et al*., 2018; Morgan *et al*., 2010; Veling *et al*., 2008). In contrast, evidence from WMH studies, including those conducted in Lebanon and Iraq in the Middle East, found that migrants were significantly less likely than non-migrants to have lifetime PEs especially hallucinatory PEs (McGrath *et al*., 2015). A migrant group may include different cultural or ethnic groups and have different migratory experiences as well as different psychotic experiences. A limitation of the current literature is that most studies have not disentangled the effects of migration and culture or ethnicity as potential risk factors for PEs.

Culture of origin or ethnicity can affect the content and characteristics of these experiences, as well as the meaning ascribed to them. This in turn can influence whether or not an experience is identified as a PE (Larøi *et al*., 2014; Murphy, 1976; Myers, 2011; Peters *et al*., 1999). For example, Kent and Wahass (1996) compared the auditory hallucinations of patients with schizophrenia in Saudi Arabia and the United Kingdom and found that the Saudi Arabian compared to British patients were more likely to describe hallucinations with religious content. Cultural beliefs may also contribute to what may be considered as ‘odd’ or ‘bizarre’ beliefs without necessarily being associated with psychotic disorders, psychological distress or psychopathology in general (Kent and Wahass, 1996; Mullen, 2003).

Qatar is a small, stable, and economically prosperous Muslim country in the Arabian Peninsula, which has undergone rapid urbanization and modernization in the past three decades. Qatar represents a unique social setting, as the majority of the population consists of migrants, on short-term contracts, from Arab and other countries from all over the world (Bel-Air, 2014). This unique social context enabled us to study the role of ethnicity by looking at migrant and non-migrant Arabs (Qataris) and comparing them with other migrant ethnicities. To the best of our knowledge, this is the first study of PEs of Arabs in a non-war zone. This exploratory study offers a novel look at both migrant status and ethnicity within the Middle East.

## Methods

Face-to-face household interviews were conducted with consenting participants who were 18 years or older living in Qatar. These interviews were conducted by the Social and Economic Research Institute (SESRI) at Qatar University as part of the Annual Omnibus survey. The survey covered a broad range of social, economic, health, and political issues.

### Sample design

The State of Qatar is divided into six administrative municipalities, 72 zones, and 320 blocks. To draw a nationally representative sample, the households in each municipality were ordered by geographic location and type of household residents – Qatari citizens (non-migrants) and resident expatriates (migrants). A probability-based sample was drawn from each stratum. Non-migrants were oversampled, as they are a minority group in the population. Inside each selected household, one eligible adult was randomly selected using a validated adaptation of the Kish method (Kish, 1965) in Qatar (Le *et al*., 2012). Weights were constructed to account for sampling disproportionality and nonresponse. The target population for this study excluded non-residential units such as army barracks, hospitals, dormitories, prisons, and camps housing blue-collar migrants.

### Sample size determination

The estimated required sample size was 1160, which was calculated using the following standard sample size formula for complex survey design (Cornfield, 1951; Kish, 1965): *n* = *z*^2^(*p*(1 − *p*)/*e*^2^)*deff*. The estimate was based on *α* value of 0.05 or *z* = 1.96; *p* or estimate of the proportion set at 50.0% to identify the largest sample size requirement, desired sampling error *e* = ±3.5%, *deff* or design effect = 1.48, and a survey response rate = 70.0%. The latter two estimates were based on prior face-to-face surveys conducted by SESRI in the same population.

### Data collection

Interviews were conducted in June of 2016 using computer-assisted personal interview technology. Interviewers and supervisors, most of whom had relevant prior survey fielding experience, were recruited and carefully trained to conduct the study interviews. During survey fielding, each sampled household was visited by a pair of interviewers (a male and a female) and a senior researcher who directly supervised the interviewers in the field. To observe traditional customs, the interviewers were matched to the respondents' gender. A total of 1356 interviews were completed, including 637 non-migrants and 719 migrants. The response rate was 64% for the former and 79% for the latter group.

### Language and translation procedures

All survey interviews were conducted either in Arabic or English. All psychological measures were independently translated from English to Arabic by the first author and another researcher. Two other independent researchers who had not seen the original English version of these measures back-translated the two Arabic versions to English. All bilingual research team members met and reviewed the two Arabic versions of the questionnaire and compared the original and back-translated English versions. Minor discrepancies in translation arose and were resolved by consensus among bilingual team members.

### Instrument

PEs were assessed using a 7-item screener adapted from the Psychosis module of the World Mental Health (WMH) Survey Initiative Version of the World Health Organization (WHO) Composite International Diagnostic Interview (CIDI) (WHO-WMH CIDI) (Kessler and Ustün, 2004). For exact questions' wording in Arabic and English, please refer to Appendix I. Briefly and without further probing, participants were asked if they ever had any of the following experiences while not having fever, dreaming, half-asleep, or under the influence of substances or drugs: (1) seeing something other people who were there could not see; (2) hearing voices that other people could not hear; (3) believing that some mysterious force was inserting strange thoughts into your head; (4) believing your thoughts were being stolen out of your mind by strange force; (5) believing your mind was being taken over by strange forces that were making you do things you did not choose to do; (6) thinking that some strange force was trying to communicate directly with you by sending special signs or signals; and (7) believing that there was an unjust plot going on to harm you or to have people follow you. ‘Yes’ or ‘No’ response options were provided. Refusals and ‘Don't Know’ were unread options and were indicated by the interviewers where appropriate.

Having ever experienced PEs (dependent variable) was defined by a positive response to at least one of the seven items, which was used to estimate the lifetime prevalence of PEs in the sample.

### Classification of participants, employment status, socio-demographics

Participants were classified into one of two migrant status groups (migrant or non-migrant) based on information about the type of household (Qataris or non-Qataris) obtained from the sampling frame and confirmation by fielding supervisors upon approaching the households for interviews.

Ethnicity or cultural groups were based on nationality, which were broadly grouped based on geographical regions into the following: Arab (Arabic speaking Middle Eastern countries: *n* = 985), South Asian (India, Bangladesh, Pakistan, Nepal, Siri Lanka: *n* = 232), Other Asian (Philippines, China, Indonesia, Iran: *n* = 39), African (Ethiopia, Senegal, Zimbabwe, Kenya, South Africa: *n* = 15), and Western (UK, US, Canada, Australia: *n* = 12). To gain more statistical power, we further collapsed non-Arab ethnicities into one category to compare with Arab ethnicity.

Migrants were asked about the month and year they arrived in Qatar, which allowed for computation of the length of stay in the country. For non-migrants and migrants who reported living in Qatar all their life, the age of respondent was used to approximate the length of duration in the country. This variable was moderately positively skewed (skewness value = 0.485) and was log transformed to restore normality before treating it as a continuous variable in statistical models.

The current employment status of non-migrants and migrants was also ascertained. Standard demographic information was also collected: age, gender, education, and marital status. To reduce non-response for income, both non-migrants and migrants were asked a series of broad questions about their total monthly income that allowed the derivation of fourteen income categories (in Qatari Riyals or QR). However, as non-migrants generally have higher income than migrants, different lower- and upper-income brackets were asked for each group. Based on these different cut-offs and the distribution of respondents in each group of these categories, the following tertiles were generated: lowest tertile of monthly income was <30k QR for non-migrants and <10k QR for migrants; middle tertile of monthly income was 30–50k QR for non-migrants and 10–20k QR for migrants; and upper tertile of monthly income was 50k QR or more for non-migrants and 20k QR or more for migrants.

Psychological distress was assessed using the Kessler 6-item scale measuring non-specific psychological distress over the last 30 days (Kessler *et al*., 2003). The scale has been previously validated in Arabic-speaking populations (Easton *et al*., 2017). As no clinically validated cut-off for this scale has been determined in Arabic speaking populations and to facilitate interpretability of the results from this scale, three ordinal levels (‘Low’, ‘Moderate’, and ‘High’) were created based on tertiles of the composite score of all six items.

Satisfaction with life in the host country was assessed using a scale from 0 to 10; all participants were asked to rate their life in Qatar, where 0 represents Qatar as the worst possible place to live and 10 represents Qatar as the best possible place to live. This variable was highly negatively skewed (skewness value = −1.711). Therefore, it was coded as a categorical variable based on the following cut-offs: category 1 (ratings between 0 and 8), category 2 (rating = 9), and category 3 (rating = 10).

Odd beliefs and magical thinking (OBMT) were assessed using the endorsement of one or more of five ‘Yes/No’ questions from the Schizotypal Personality Questionnaire (Raine, 1991). These questions related to: (1) belief in telepathy (mind-reading); (2) belief in one's ability to communicate with others telepathically; (3) belief in clairvoyance (psychic forces, fortune telling); (4) belief that others can tell what you are thinking; and (5) belief in having experiences with seeing the future, paranormal (psychic) powers or the sixth sense. It was necessary to control for items that index OBMT in the analysis because these items may be more likely to be endorsed in certain cultures than others outside of the context of psychopathology (Mullen, 2003). OBMT may also index trait-like susceptibility for schizophrenia and psychotic disorder in general, and as such may also be independently associated with PEs (Kwapil *et al*., 2013; Miller *et al*., 2002; Walter *et al*., 2016). Therefore, it was important to control for OBMT in our analysis as it could potentially confound the association between ethnicity and PEs

### Statistical analyses

Initial bivariate analyses were conducted using the χ^2^ test of proportions to compare lifetime prevalence estimates of PEs by ethnicities and other socio-demographics across migrants and non-migrants. To correct for survey design effects on the sampling variances of these proportions, the F-transformed version of the Pearson χ^2^ statistic was used (Heeringa, West and Berglund, 2011).

Univariate and multivariate logistic regression models were fitted to assess the effect of ethnicity on the lifetime prevalence of PEs before and after adjustment for migration-related variables, socio-demographics, psychological distress, and OBMT. Hierarchal adjustment for socio-demographics, ethnicity, psychological distress, OBMT and PEs were also conducted to examine the pattern of co-variation between these variables and PEs.

By fitting main effects of all variables and interaction between psychological distress (three categorical levels) and ethnicity (Arab *v.* non-Arab), we tested for potential moderation by psychological distress on the association between ethnicity and PEs. The design-adjusted Wald test was used to assess the goodness of fit by comparing models with and without these interaction terms. Two-tailed *p*-values <0.05 were considered statistically significant.

As the literature is still unclear whether schizotypy and PEs index the same phenotype or measure different, but overlapping phenotypes (Debbane and Barrantes-Vidal, 2015; Torgersen, 1985); we conducted a sensitivity analysis that assumed both constructs index the same phenotype and calculated a composite severity score that captured the distribution of the sample on the total number of psychotic experiences and OBMT items endorsed. For this part of the analysis, an ordinal logistic regression model was fitted with symptom severity score as the dependent variable; model assumptions and fit to data were verified and evaluated. Previous analysis was re-run using the same set of variables with the exception of OBMT, which comprised part of the dependent variable in this segment of the analysis.

All statistical analyses were weighted and carried out in STATA version 13 (StataCorp, 2013).

## Results

Sample characteristics and distribution of potential determinants of PEs by ethnicity and in the entire sample are presented in Table 1.

**Table 1.** Socio-demographics and other characteristics by Arab ethnicity and in general population sample

Variables	Variable levels	Arab (*n* = 1023)			Non-Arab (*n* = 330)			Total sample (*n* = 1353)		
		*n*/*N*	%	95% CI	*n*/*N*	%	95% CI	*n*/*N*	%	95% CI
Gender	Female	400/1023	49.2	45–53.4	165/330	50.5	44.3–56.7	565/1353	49.6	46.0–53.1
	Male	623/1023	50.7	43.3–55.7	165/330	49.5	43.3–55.7	788/1353	50.4	46.9–54.0
Age (years)	18–24	97/1006	13.4	10.6–16.7	9/327	2.8	1.3–6.0	106/1333	9.4	74.9–11.6
	25–34	246/1006	25.7	22.2–29.5	107/327	30.0	24.7–35.9	353/1333	27.3	24.3–30.5
	35–44	295/1006	27.0	23.5–30.9	137/327	41.6	35.6–47.8	432/1333	32.7	29.4–36.1
	45+	368/1006	33.9	30.0–38.0	74/327	25.5	20.3–31.6	442/1333	30.7	27.5–34.1
Marital status	Single	182/1022	19.7	16.6–23.2	17/329	5.5	32.1–93.2	199/1351	14.2	12.0–16.8
	Married	840/1022	80.3	76.8–83.4	312/329	94.5	90.7–96.8	1152/1351	85.8	83.3–88.0
Migrant status	Non-migrant	637/1023	40.3	37.6–43.1	0/0	–	–	637/1353	24.5	23.1–26.0
	Migrant	386/1023	59.7	56.8–62.4	330/330	–	–	716/1353	75.5	74.0–77.0
Formal education	Yes	794/1007	82.7	79.6–85.5	306/328	94.5	90.9–96.8	1110/1335	87.4	85.1–89.4
	No	213/1007	17.2	14.5–20.4	22/328	5.5	3.2–9.1	235/1335	12.6	10.6–14.9
Employment status	Unemployed	393/1012	43.0	38.8–47.2	111/322	34.0	28.4–40.0	504/1334	39.3	36–48.8
	Employed	619/1012	57.1	52.9–61.2	211/322	66.0	60–71.6	830/1334	60.7	57.2–64.0
Household income categories^a^	Lowest	130/303	45.3	37.8–53.0	55/131	40.1	31.1–49.8	185/434	43.0	37.2–49.1
	Middle	102/303	34.0	27.0–41.7	46/131	36.0	27.3–45.6	148/434	34.8	29.3–40.8
	Highest	71/303	20.7	15.4–27.3	30/131	23.9	16.4–33.5	101/434	22.1	17.5–27.6
Psychological distress in the past 30 days^b^	Lowest	356/1016	35.0	31.1–39.2	179/320	58.0	51.8–64.1	535/1336	43.4	40.5–47.5
	Middle	307/1016	29.1	25.5–33.0	64/320	19.9	15.3–25.5	371/1336	25.5	22.6–28.6
	Highest	353/1016	35.9	31.9–40.0	77/320	22.1	17.3–27.7	430/1336	30.6	27.4–33.9
Satisfaction rating with living in Qatar	0–8	627/1023	55.9	51.7–60.0	96/330	25.0	20.1–30.6	723/1353	43.8	40.5–47.1
	9	159/1023	15.7	12.9–19.0	65/330	20.4	15.8–25.9	224/1353	17.6	15.1–20.5
	10	237/1023	28.4	24.7–32.5	169/330	54.6	48.4–60.7	406/1353	38.6	35.3–42.1
Odd belief or magical thinking	Yes	457/1020	43.2	39.1–47.4	208/327	66.3	60.3–71.9	665/1347	52.1	48.6–55.6
	No	563/1020	56.8	52.6–60.9	119/327	33.7	28.1–39.7	682/1347	47.9	44.4–51.4

All proportions are weighted. Total sample is 1353 and includes everyone who responded to at least one question in the Psychotic Experiences module and who reported on ethnicity

a Lowest tertile of monthly income is <30k Qatari Riyals for non-migrants (Qataris) and <10k Qatari Riyals for migrants. Middle tertile of monthly income 30–50k Qatari Riyals for non-migrants (Qataris) and 10–20k Qatari Riyals for migrants. Highest tertile of monthly income is 50k+ Qatari Riyals for non-migrants (Qataris) and 20k+ Qatari Riyals for migrants.

b Lowest tertile of psychological distress (K6) corresponds to a score range of 0 to <8. Middle tertile of psychological distress (K6) corresponds to a score range of 8 to 9. Highest tertile of psychological distress (K6) corresponds to a score range of >9 to 24.

The lifetime prevalence of PEs was 27.9% (Table 2). Visual hallucinations were the most commonly reported (12.8%) followed by delusions of thought interference (10.8%), thought passivity (10.2%), and mind control (9.5%). Auditory hallucinations (6.9%) and persecutory or paranoid delusions (6.7%) were less prevalent, while ideas of reference (3.6%) were the lowest reported symptom in this population (Table 2).

**Table 2.** Lifetime prevalence of psychotic symptoms and symptom profiles by ethnicity and in total sample

	Arab		Non-Arab		*p* value	Total sample	
	Prevalence % (*n*/*N*)	95% CI	Prevalence % (*n*/*N*)	95% CI		Prevalence % (*n*/*N*)	95% CI
Symptom types^a^							
Visual hallucinations	15.5 (152/999)	12.7–18.7	8.6 (27/311)	5.7–12.8	0.008	12.8 (179/1310)	10.7–15.3
Auditory hallucinations	8.8 (89/998)	6.6–11.6	3.8 (15/310)	2.2–6.7	0.007	6.9 (104/1308)	5.4–9.0
Thought interference (thought insertion)	13.9 (121/997)	11.2–17.2	5.7 (19/312)	3.4–9.2	<0.001	10.8 (140/1309)	8.8–13.1
Thought passivity (thoughts are being broadcasted/stolen)	13.1 (117/995)	10.4–16.3	5.6 (20/313)	3.5–9.0	0.001	10.2 (137/1308)	8.3–12.5
Mind control (mind taken over by strange forces)	12.4 (109/993)	9.8–15.5	4.7 (15/313)	2.7–8.0	<0.001	9.5 (124/1306)	7.6–11.7
Ideas of reference (strange forces trying to communicate directly with you)	4.4 (52/989)	3.0–6.3	2.3 (8/310)	1.0–5.0	0.134	3.6 (60/1299)	2.5–5.0
Persecution or following (plot to harm you or people following you)	7.8 (76/995)	5.8–10.4	4.9 (15/315)	2.8–8.6	0.155	6.7 (91/1310)	5.1–8.7
Any psychotic experiences^b^	34.7 (324/985)	30.7–38.9	16.4 (51/298)	12.2–21.8	<0.001	27.9 (375/1283)	24.8–31.2
Number of symptoms reported mean (s.d.)	2.2 (1.4)	2.0–2.3	1.8 (1.2)	1.5–2.1	0.042	2.1 (1.4)	1.9–2.2
Symptom profiles^b^							
Hallucinations only	6.4 (75/985)	5.5–9.9	7.4 (19/298)	3.9–10.4	0.632	7.0 (94/1283)	5.5–9.2
Delusions only	14.6 (132/985)	11.7–17.9	6.7 (20/298)	4.0–10.9	0.003	11.6 (152/1283)	9.5–14.1
Mixed hallucinations and delusions	12.8 (117/985)	10.2–15.9	3.3 (12/298)	1.7–6.3	<0.001	9.3 (129/1283)	7.4–11.4

All proportions are weighted.

a Estimates for symptom types are based on respondents who may or may not complete the entire Psychotic Experiences module.

b Estimates for any psychotic experiences and symptom profiles are based only on respondents who completed the entire PEs module and who reported on ethnicity.

Lifetime prevalence estimates of any PEs were significantly higher in Arabs (34.7%) compared with non-Arabs (16.4%) across all symptoms (*p* < 0.001) with the exception of ideas of reference and paranoid delusions (Table 2).

As also shown in Table 2, symptom profiles in the entire sample were largely marked by delusions only (11.6%) and to less extent by hallucinations only (7.7%). An appreciable proportion of the population reported experiencing a mixture of hallucinations and delusions (9.3%). While similar proportions of Arabs (6.4%) and non-Arabs (7.4%) reported experiencing hallucinations only (*p* = 0.632), Arab respondents reported significantly higher delusions only (14.6% *v.* 6.7%, *p* = 0.003) and higher delusions with hallucinations compared to non-Arabs (12.8% *v.* 9.8%, *p* < 0.001).

Table 2 presents lifetime prevalence estimates of PEs in the entire sample by socio-demographic characteristics and reported levels of psychological distress in the past 30 days. The prevalence of PEs was generally higher in non-migrant groups (*p* < 0.001). Arab participants exhibited higher prevalence of PEs compared to other ethnicities (*p* < 0.001). The prevalence of PEs was significantly higher in females than males (*p* < 0.001), single compared to married respondents (*p* < 0.001), and those with no formal education compared to those with formal education (*p* = 0.028). While no significant variations were observed in the prevalence of PEs by income levels (*p* = 0.096), respondents who were unemployed reported a significantly higher prevalence of PEs than those who were employed (*p* = 0.003). The highest prevalence of PEs was observed in the youngest age group (18–24 years of age) and there was a general trend of decreasing prevalence with increasing age (*p* = 0.002). The prevalence of PEs was observed to significantly increase with increasing levels of reported psychological distress (*p* < 0.001).

Prevalence estimates of PEs by socio-demographics and other potential risk factors were further explored within ethnic Arab and non-Arab groups as shown in Table 2. The prevalence of PEs did not differ between migrant and non-migrant Arabs (*p* = 0.123). Female gender was associated with a higher prevalence of PEs in the Arab group only (*p* < 0.001). The trend of decreasing prevalence of PEs with increasing age was statistically significant in the non-Arab group only (*p* = 0.002). Formal education was associated with lower prevalence of PEs in the non-Arab group only (*p* < 0.001).

Unemployment status was only associated with a higher prevalence of PEs in the Arab group (*p* = 0.014). Psychological distress and odd beliefs or magical thinking were consistently and significantly (*p* < 0.001) associated with a higher prevalence of PEs in both groups (see Table 2). Notably, the prevalence of PEs was significantly higher among Arab respondents who held OBMT compared to those who did not hold these beliefs (48.8% *v.* 15.8%, *p* < 0.001). A similar observation was also made in non-Arabs (35.2% *v.* 7.3%, *p* < 0.001)

The results from univariate and multivariate models are presented in Table 3. Of all the socio-demographic variables, Arab ethnicity was the only variable that remained significantly associated with PEs after simultaneous adjustment for all other variables including OBMT as shown in Model 2 (OR = 2.10, *p* = 0.015). Model 1 controlled for all variables except OBMT. In this model, only the highest level of psychological distress compared to the lowest level of distress was significantly associated with PEs (OR = 3.50, *p* < 0.001). This estimate of association was further reduced (OR = 2.29), but remained statistically significant (*p* = 0.003) after adjustment for OBMT (Model 2). OBMT was strongly associated with PEs before (OR = 6.31, *p* < 0.001) and after adjustment for all variables (OR = 6.25, *p* < 0.001).

**Table 3.** Frequencies for socio-demographics and other characteristics of Arab and non-Arab subpopulations for associations with a lifetime prevalence of any psychotic experiences (PEs)

Variables		Prevalence estimates of PEs								
		Arab (*n* = 985)			Non-Arab (*n* = 298)			Total sample (*n* = 1283)		
		% (*n*/*N*)	95% CI	Χ^2^ *p* value	% (*n*/*N*)	95% CI	Χ^2^ *p* value	% (*n*/*N*)	95% CI	Χ^2^ *p* value
Migrant status	Non-migrant	38.4 (192/612)	33.4–43.7	0.123	–	–	–	38.4 (192/612)	33.4–43.7	<0.001
	Migrant	32.2 (132/373)	26.7–38.3		16.4 (51/298)	12.2–21.8		24.4 (183/671)	20.7–28.4	
Gender	Female	43.1 (174/388)	36.9–49.5	<0.001	20.4 (30/144)	13.9–28.9	0.098	34.6 (204/532)	29.8–39.7	<0.001
	Male	26.4 (150/597)	21.7–31.8		12.5 (21/154)	7.7–19.5		21.2 (171/751)	17.6–25.4	
Age (years)	18–24	43.5 (40/97)	31.8–56.0	0.161	62.2 (3/8)	24.5–89.3	0.002	45.5 (43/105)	34.6–58.0	<0.001
	25–34	37.9 (87/237)	30.2–46.4		21.6 (20/97)	13.4–32.8		31.2 (107/334)	25.3–37.8	
	35–44	28.8 (91/289)	22.2–36.4		15.2 (20/126)	9.5–23.6		22.3 (111/415)	17.6–27.7	
	45+	33.3 (103/349)	27.0–41.3		8.0 (8/64)	3.5–17.1		26.0 (111/413)	20.6–32.0	
Marital status	Single	45.7 (77/177)	36.2–55.4	0.010	43.1 (7/17)	19.8–69.9	0.008	45.3 (84/194)	36.3–54.5	<0.001
	Married	32.1 (247/807)	27.8–36.7		14.7 (44/280)	10.6–20.0		24.9 (291/1087)	21.8–28.4	
Formal education	No	33.4 (68/204)	25.2–42.8	0.819	51.2 (10/20)	25.1–76.6	<0.001	36.3 (78/224)	28.0–45.6	0.028
	Yes	34.6 (249/767)	30.2–39.4		14.1 (40/276)	10.1–19.3		26.3 (289/1043)	23.0–29.8	
Employment status	Unemployed	40.5 (159/379)	34.2–47.1	0.014	19.0 (18/100)	11.7–29.4	0.436	33.7 (177/479)	28.6–39.2	0.003
	Employed	30.0 (162/596)	25.1–35.5		15.0 (32/194)	10.1–21.6		23·9 (194/790)	20.2–28.1	
Income categories^a^	Lowest	31.2 (75/211)	23.4–40.2	0.120	25.4 (16/69)	15.1–39.4	0.007	29·0 (91/280)	22.6–36.5	0.094
	Middle	40.9 (93/246)	32.9–49.4		15.3 (18/103)	9.1–24.6		30.0 (111/349)	24.3–36.4	
	Highest	30.6 (98/328)	24.2–37.9		5.9 (8/90)	2.6–13.0		21.7 (106/418)	17.1–27.1	
Psychological distress (K6) in the past 30 days^b^	Lowest	24.3 (156/342)	18.7–30.9	<0.001	8.7 (23/71)	4.9–15.0	<0.001	16.7 (100/508)	13.0–20.9	<0.001
	Moderate	29.6 (81/294)	23.0–37.1		23.4 (13/59)	13.3–37.7		27.7 (94/353)	22.0–34.3	
	High	49.0 (86/345)	41.8–56.3		30.6 (14/163)	19.4–44.7		44.2 (179/413)	38.0–50.9	
Satisfaction rating with living in home or host country	0–8	36.7 (194/608)	31.5–42.2	0.424	22.8 (18/82)	13.5–35.8	0.187	34.1 (212/690)	29.2–39.0	0.004
	9	29.0 (49/150)	20.6–39.1		19.4 (13/61)	11.0–32.1		24.5 (62/211)	18.3–32.4	
	10	33.8 (81/227)	26.2–42.4		12.6 (20/155)	7.8–19.8		22.3 (101/382)	17.7–27.9	
Odd beliefs or magical thinking	Yes	48.8 (251/547)	43.1–4. 5	<0.001	35.2 (36/107)	25.3–46.5	<0.001	45.3 (287/654)	40.3–50.4	<0.001
	No	15.7 (73/438)	11.6–1.0		7.3 (15/191)	4.1–12.5		11.6 (88/629)	8.8–15.2	

All proportions are weighted.

a Lowest tertile of monthly income is <30k Qatari Riyals for non-migrants (Qataris) and <10K Qatari Riyals for migrants. Middle tertile of monthly income 30–50k Qatari Riyals for non-migrants (Qataris) and 10–20k Qatari Riyals for migrants. Highest tertile of monthly income is 50k+ Qatari Riyals for non-migrants (Qataris) and 20k+ Qatari Riyals for migrants.

b Lowest tertile of psychological distress (K6) corresponds to a score range of 0 to <8. Middle tertile of psychological distress (K6) corresponds to a score range of 8 to 9. Highest tertile of psychological distress (K6) corresponds to a score range of >9 to 24.

In online Supplementary Table S1, we present models with hierarchal adjustment of the same covariates. In a model that adjusts for all standard socio-demographic variables except for Arab ethnicity (Model 1), migrant status (OR = 0.55, *p* = 0.003), female gender (OR = 2.05, *p* = 0.002), single *v.* married status (OR = 2.01, *p* = 0.017), and middle income *v.* highest income category (OR = 1.72, *p* = 0.015) were the only socio-demographic variables that were significantly associated with PEs.

In the next model (Model 2) that further adjusts for ethnicity (OR = 2.69, *p* < 0.001), migrant status was no longer statistically associated with PEs (OR = 0.79, *p* = 0.285). However, gender (OR = 2.00, *p* = 0.002), marital status (OR = 1.91, *p* = 0.038), and middle income *v.* highest income category (OR = 1.70, *p* = 0.018) were still significantly associated with PEs.

In the next model (Model 3) that further adjusts for psychological distress (online Supplementary Table S1), all associations with socio-demographic variables including Arab ethnicity (OR = 2.24, *p* = 0.002), gender (OR = 1.72, *p* = 0.021), middle income (OR = 1.60, *p* = 0.038) were further reduced and the association between marital status and PEs were no longer statistically significant (OR = 1.81, *p* = 0.054). Notably, only the highest tertile of psychological distress *v.* lowest tertile comparison (OR = 3.26, *p* < 0.001) was significantly associated with PEs.

In the next model (Model 4) that further adjusts for OBMT (OR = 5.21, *p* < 0.001), the association between female gender and PEs was further reduced and were no longer statistically significant (OR = 1.29, *p* = 0.312). The middle income *v.* highest income comparison for PEs was borderline significant (OR = 1.60, *p* = 0.045). Meanwhile, Arab ethnicity remained significantly associated with PEs (OR = 2.24, *p* = 0.002). The association of higher level of psychological distress with PEs was further reduced upon adjustment of OBMT (OR = 2.21), but remained statistically significant (*p* = 0.003).

We found no evidence that the association between ethnicity and PEs differed by levels of psychological distress (ethnicity × moderate *v.* low levels of distress: OR = 0.28, *p* = 0.056; ethnicity × high *v.* low levels of distress: OR = 0.51, *p* = 0.306). Additionally, the adjusted Wald test did not suggest that the inclusion of these interaction terms improved the fit of the model (*F*-statistic = 1.94, *p* = 0.145). Furthermore, the main effects of both ethnicity (OR = 4.77, *p* = 0.002) and psychological distress (moderate *v.* low levels of distress: OR = 4.15, *p* = 0.017; high *v.* low levels of distress: OR = 5.76, *p* = 0.004) remained statistically significant even after the inclusion of these interaction terms in the model.

Similar results to those presented for Model 1 in Table 4 were obtained upon repeating the analysis with the dependent variable defined using a composite severity score of PEs and OBMT. Furthermore, there was no evidence of moderation by psychological distress on the association between ethnicity and severity score for PEs and OBMT. This gave us confidence that our findings were robust irrespective of the way our dependent variable was operationalized (experiencing any PEs *v.* composite severity score of PEs and OBMT).

**Table 4.** The association between ethnicity, migration-related variables, socio-demographics, psychological distress, and psychotic experiences

Variables		Univariate models^a^			Model 1^b^			Model 2^c^		
		OR	95% CI	*p* value	OR	95% CI	*p* value	OR	95% CI	*p* value
Ethnicity	Arab	2.70	1.83–4.00	<0.001	2.67	1.49–4.78	0.001	2.10	1.15–3.82	0.015
	Non-Arab	ref	–		ref	–		ref	–	–
Migrant status	Migrant	0.52	0.38–0.70	<0.001	0.85	0.45–1.60	0.609	0.91	0.48–1.76	0.788
	Non-migrant	ref	–		ref	–		ref	–	–
Gender	Female	1.96	1.42–2.70	<0.001	1.66	1.03–2.68	0.038	1.18	0.70–1.98	0.529
	Male	ref	–		ref	–		ref	–	
Age (years)	18–24	2.45	1.39–4.30	0.002	0.97	0.56–1.66	0.903	1.00	0.40–2.48	0.993
	25–34	1.30	0.86–1.98	0.217	1.18	0.67–2.08	0.576	1.13	0.60–2.11	0.703
	35–44	0.82	0.54–1.25	0.356	0.99	0.42–2.36	0.987	0.86	0.49–1.52	0.613
	45+	ref	–	–	ref	–	–	ref	–	–
Marital status	Single	2.54	1.68–3.83	<0.001	1.87	1.00–3.48	0.048	1.52	0.78–2.96	0.216
	Married	ref	–		ref	–		ref	–	–
Formal education	No	1.60	1.05–2.44	0.029	0.74	0.39–1.40	0.352	0.87	0.44–1.73	0.698
	Yes	ref	–		ref	–		ref	–	–
Employment status	Unemployed	1.61	1.17–2.23	0.004	1.16	0.71–1.91	0.546	1.25	0.72–2.16	0.429
	Employed	ref	–		ref	–		ref	–	–
Income categories^d^	Lowest	1.47	0.93–2.30	0.096	1.07	0.61–1.87	0.823	1.17	0.66–2.06	0.584
	Middle	1.55	1.02–2.35	0.038	1.59	1.00–2.50	0.047	1.60	0.97–2.64	0.068
	Highest	ref	–	–	ref	–	–	ref	–	–
Psychological distress in the past 30 days^e^	High	4.02	2.72–5.93	<0.001	3.50	2.11–5.81	<0.001	2.29	1.33–3.95	0.003
	Moderate	1.93	1.27–2.95	0.002	1.56	0.90–2.72	0.114	1.19	0.66–2.14	0.569
	Low	ref	–	–	ref	–	–	ref	–	–
Length of stay in Qatar	Log of duration in years	1.26	1.07–1.48	0.006	1.02	0.75–1.39	0.887	1.07	0.78–1.47	0.666
Satisfaction rating of host country	0–8	0.63	0.41–0.97	0.038	1.03	0.58–1.82	0.911	1.03	0.56–1.91	0.920
	9	0.57	0.39–0.80	0.002	0.74	0.41–1.32	0.312	0.82	0.46–1.47	0.499
	10	ref	–	–	ref	–	–	ref	–	–
Odd beliefs or magical	Yes	6.31	4.36–9.12	<0.001	–	–	–	6.25	3.87–10.11	<0.001
	No	ref	–	–	ref	–	–	ref	–	–

Odds ratio (OR) and corresponding 95% confidence intervals (95% CI). Reference group (ref) is the comparison group. All models are weighted.

a The unadjusted OR from a univariate model of each of the variables and its association with psychotic experiences are presented.

b Model 1 (*n* = 940) presents the adjusted ORs controlling for most variables simultaneously without adjustment for odd beliefs and magical thinking.

c Model 2 (*n* = 940) presents the adjusted ORs controlling for all variables including adjustment for odd beliefs and magical thinking.

d Lowest tertile of monthly income is <30k Qatari Riyals for non-migrants (Qataris) and <10k Qatari Riyals for migrants; middle tertile of monthly income 30–50k Qatari Riyals for non-migrants (Qataris) and 10–20k Qatari Riyals for migrants; highest tertile of monthly income is 50k+ Qatari Riyals for non-migrants (Qataris) and 20k+ Qatari Riyals for migrants.

e Lowest tertile of psychological distress (K6) corresponds to a score range of 0 to <8; middle tertile of psychological distress (K6) corresponds to a score range of 8 to 9; highest tertile of psychological distress (K6) corresponds to a score range of >9 to 24.

## Discussion

The proportion of the general population that reported experiencing any psychotic symptom at some point in their life was 27.9%, which is relatively high for a war-free country like Qatar. A similar lifetime prevalence estimate (28.4%) was reported in the National Comorbidity Study of the USA (Kendler *et al*., 1996) and both estimates are quite high compared to the 23.3% estimate obtained using the MINI in a conflict-afflicted population of South Sudan (Ayazi *et al*., 2016). The rapid rate of urbanization and immigration that Qatar experienced over the past three decades may contribute to the relatively high prevalence we report. Urbanicity and migration are known risk factors for PEs and psychosis (Binbay *et al*., 2012; Broome *et al*., 2005; Goldstein and Rodnick, 1975; Pedersen and Mortensen, 2001; van Os, 2004). In a UK population, Johns *et al*. (2004) reported PEs in 65.9% in urban *v.* 34.1% in a rural population (Johns *et al*., 2004). In contrast, CIDI-based lifetime prevalence estimates of PE as low as 3.6% were reported for a highly urban city in Turkey (Alptekin *et al*., 2009).

The most unique finding from our study pertains to the role of ethnicity on potential risk of PEs independent of migration. Qatar is a unique social setting as the majority of the population is composed of migrants of multi-ethnicity. Our lifetime prevalence estimate was significantly higher for Arabs than non-Arabs and did not significantly differ between Arab migrants and non-migrants, but was substantially lower for non-Arab migrants. Migration was not a factor in determining PEs in our sample, which is consistent with findings from a recent meta-analysis (Leaune *et al*., 2018), but counter to some studies that indicate migration, social adversity, and negative social experience may be cumulative, particularly in migrant groups, in predisposing to PEs (Bourque, Ven, and Malla, 2011) and psychosis (McKenzie *et al*., 2008). The context of where the migrant moves to is important, as the high rates of PEs and psychosis reported in second-generation immigrants has been attributed to psychosocial adversity (e.g. victimization) in ethnic minority groups (Bourque, Ven and Malla, 2011; Johns *et al*., 2004; McKenzie *et al*., 2008). In a 20-year follow up population study, alienation and paranoid ideation was prevalent, the highest (58%) ‘having ideas that others do not share’ as would be the case in groups without peer support from those with a shared cultural background (Rössler *et al*., 2007). Our findings indicate that non-Arab migrants are not more vulnerable to PEs than the indigenous Qatari population, and there was no difference in satisfaction ratings of living in Qatar between the groups.

The prevalence of PEs in the Arab population was increased across all symptom types and included hallucinations in both visual and auditory modalities, thought interference, passivity and ideas of mind control. These are first-rank symptoms of schizophrenia that would be difficult to imagine if not actually experienced by individuals, which adds to the veracity of the findings. We found no ethnicity difference in ideas of reference or paranoid ideation as distinct from studies that indicate the presence of paranoia in those ethnic minorities who migrate to other countries (McKenzie *et al*., 2008). In addition, we found the prevalence of delusions and those mixed with hallucinations was higher in Arabs than non-Arabs in general. Higher prevalence of delusions especially those with more unusual delusional ideas (e.g. thought interference as opposed to persecutory delusions) are more likely to be of clinical significance and less likely due to ‘real life’ experiences (Freeman, 2006). It is important to note that delusions are often associated with hallucinatory experiences in psychosis (Krabbendam *et al*., 2004). Therefore, the high prevalence of this mixed profile of delusional ideation among Arabs compared to non-Arabs further support the potential clinical significance of these symptoms in this subset of the population. Our findings are also consistent with a recent meta-analysis that found participants from the Maghreb and the Middle East were at greatest risk for psychotic symptoms (OR 3.30, 95% CI 2.09–5.21) compared with Asian, Black, and White ethnic groups (Leaune *et al*., 2018).

It is important to note that significantly higher proportions of Arabs reported odd (paranormal) beliefs and magical thinking than non-Arabs, including beliefs in clairvoyance, mind reading, and the ability to communicate with others telepathically as well as beliefs in paranormal (psychic) experiences or the sixth sense. Ethnicity was the only socio-demographic variable that remained statistically significantly associated with PEs after adjustment for these beliefs. In Western literature, these beliefs constitute one of the five domains of schizotypal personality (Raine, 1991) and their status as a vulnerability trait for PEs has been subject to debate (Lenzenweger, 2006; Mata *et al*., 2003; Meehl, 1962). For example, Yung and Lin (2016) have suggested that paranormal and magical thinking may not be necessarily maladaptive and may mark benign PEs in the general population (Yung and Lin, 2016). In contrast, magical ideation may be more indicative of vulnerability toward psychosis if concurrent with PEs that is associated with distress and poor functioning (Yung *et al*., 2009).

It may be of interest for future studies to identify culturally driven beliefs and traits that could determine the trajectory of PEs to illness among non-clinical populations in the Arab context. For example, the extent that OBMT in this population overlaps with other unmeasured Arabic cultural idioms related to beliefs of influence or control as a result of the ‘evil eye’, evil spirits or Jinn, and bewitchment is unknown. Peters *et al*. (2017) in an attempt to determine what influenced people with PEs to seek treatment, found that a ‘clinical group’ had more threatening PEs than a ‘non-clinical’ group, who expressed more supernatural explanations. They argued that supernatural explanations were a protective mechanism against seeking treatment in those with PEs (Peters *et al*., 2016, 2017).

We also found a strong association between PEs and higher levels of psychological distress in the past 30 days. Recent stressful life events and traumatic events associated with psychological distress are strongly associated with PEs (Johns *et al*., 2004; McGrath *et al*., 2017). In our study, psychological distress was strongly associated with PEs in both Arab and non-Arab groups indicating that these symptoms, although occurring in a non-clinical population impede a sense of wellbeing. OBMT were also strongly associated with experiencing psychological distress and with PEs suggesting that these phenomena are not culturally benign. Furthermore, controlling for this trait resulted in a weakening of the association between higher levels of psychological distress and PEs, which could potentially mean that the association between psychological distress and PEs may be mediated by magical ideation and beliefs in the paranormal in this population. This is consistent with findings from a recent study that supported the role of odd beliefs in the maintenance of psychological functioning in relation to PEs (Unterrassner *et al*., 2017).

Consistent with other studies of PEs in non-clinical populations (McGrath *et al*., 2015), we found that PEs were more common in females than males. Whether females with a lifetime prevalence of PEs are at higher risk for developing psychotic disorders than females or males without these experiences is an important question for future research. Also consistent with previous studies (Bora and Arabaci, 2009; Raine, 1992; Unterrassner *et al*., 2017), female gender was strongly associated with OBMT in our study such that controlling for this trait greatly reduced the magnitude of the association between gender and PEs. This suggests that beliefs in magic and the paranormal may potentially mediate the association between female gender and PEs. The extent that this trait constitutes a vulnerability trait for PEs and psychotic disorders among females can be explored in future investigations.

In our study, marriage was negatively associated with PEs, which is also consistent with findings from previous studies (McGrath *et al*., 2015). This is perhaps as a result of the mutual support marriage provides, or that those with psychological problems may be less likely to marry or stay married. PEs are not generally thought to be associated with poverty (Read, 2010). Consistent with this, compared to the highest household income, the lowest household income category was not significantly associated with PEs in our study. However, the middle household income category was significantly associated with PEs. As this group included a disproportionate concentration of migrants reporting higher rates of unemployment (the majority of whom were unemployed housewives of working husbands), it may be that a factor in this association of middle income and PEs was relatively less social support that is often provided through employment.

### Limitations

The present study has important public health implications for rapidly developing countries such as Qatar. Limitations of this study include the following. The full population of Qatar was not surveyed as non-residential units, such as army barracks, hospitals, dormitories, prisons, and camps housing blue-collar migrants, were excluded. The latter group is hard to access population in Qatar as most live in camps owned by their employers and could potentially be more vulnerable to PEs than other household-based migrants in Qatar. In addition, blue-collar migrants are also more wary of participating in health research than any other migrant groups because of fear of deportation by their employer if any underlying physical or mental disorder is identified. Future studies of PEs in this group should be designed with careful consideration of the best way to reach this understudied segment of Qatar's general population.

English is not the mother tongue of most migrants in Qatar. As such our estimates of PEs may have been biased toward the null for migrants whose comprehension of the English language is limited.

Although interviewers were thoroughly trained in general interviewing techniques and in administering questions of sensitive nature, we cannot rule out other sources of response bias in Arab and non-Arab migrants for job security reasons if any underlying mental disorder is identified.

Our assessment of PEs was of necessity, brief. We did not apply any exclusion criteria based on personal or family history of mental illness or available diagnosis. We did not assess timing of these events in relation to other events in the person's life. We also did not assess frequency or persistence of these events or level of distress directly caused by these experiences. For delusions, we did not assess the level of conviction, which would have allowed us to tap into severity level of delusional ideation. We did not record personal history of substance misuse (cannabis and alcohol), which is strongly associated with PEs and psychosis (Compton, Chien and Bollini, 2009; Johns *et al*., 2004; Rössler *et al*., 2007). We also cannot determine the cause or effect of some key factors, e.g. psychological distress and PEs, due to the cross-sectional nature of the study.

### Future directions

Studying healthy people with PEs may help identify resilience and vulnerability, and answer the question as to why some people with PEs remain healthy (Rossell *et al*., 2014). As many with PEs have no clinical illness, it is often of interest to identify determinants of the trajectory of PEs to illness. Some factors may be culturally influenced like beliefs in paranormal phenomena; these are of prime importance in understanding vulnerability in different population groups. As we understand more about PEs and their significance in multicultural contexts, we may be in a better position to focus attention on those who are likely to need early assessment and care at an early stage in order to prevent transition to clinical mental illness in different cultural settings.

## Conclusion

Here we present the first study of prevalence of PEs in Qatar, where prevalence rates are relatively high, both in the indigenous and migrant Arab population. We also report that these experiences were significantly associated with psychological distress and OBMT in the general population of Qatar. Evidence linking Arab ethnicity, female gender, and psychological distress to PEs through associations with OBMT was identified. Future studies could prospectively investigate these associations to shed light on how PEs may increase the risk of developing psychotic disorders in these populations.

## Supplementary material

For supplementary material accompanying this paper visit http://dx.doi.org/10.1017/S0033291719000977. click here to view supplementary material
