# Supplementary material for: Lifetime prevalence and potential determinants of psychotic experiences in the general population of Qatar
Source: Psychol Med. 2019 May 28;50(7):1110–20. doi: 10.1017/S0033291719000977 (PMC7253618; doi:10.1017/S0033291719000977)
Supplement: Supplementary file 1 [file S0033291719000977sup.zip › S0033291719000977sup001.docx]

| **Supplementary Table 1: Hierarchical Logistic Regression for the Association between Ethnicity and Psychotic Experiences** | | | | | | | | | | | | | |
| --- | --- | --- | --- | --- | --- | --- | --- | --- | --- | --- | --- | --- | --- |
| **Variables** | | **Model 1** | | | **Model 2** | | | **Model 3** | | | **Model 4** | | |
|  |  | **OR** | **95% CI** | **p value** | **OR** | **95% CI** | **p value** | **OR** | **95% CI** | **p value** | **OR** | **95% CI** | **p value** |
| **Ethnicity** | Arab | --- | --- | --- | 2.70 | 1.64 – 4.41 | < 0.001 | 2.24 | 1.34 – 3.75 | 0.002 | 1.87 | 1.11 – 3.17 | 0.019 |
|  | Non-Arab | --- | --- |  | ref | --- |  | ref | --- |  | ref | --- | --- |
| **Migrant Status** | Migrant | 0.55 | 0.34 – 0.81 | 0.003 | 0.79 | 0.51 – 1.22 | 0.285 | 0.80 | 0.51 – 1.26 | 0.337 | 0.86 | 0.53 – 1.40 | 0.547 |
|  | Non-Migrant | ref | --- |  | ref | --- |  | ref | --- |  | ref | --- |  |
| **Gender** | Female | 2.04 | 1.31 – 3.18 | 0.002 | 2.0 | 1.28 – 3.13 | 0.002 | 1.72 | 1.09 – 2.73 | 0.021 | 1.29 | 0.79 – 2.10 | 0.312 |
|  | Male | ref | --- |  | ref | --- |  | ref | --- |  | ref | --- | --- |
| **Age (Years)** | 18 – 24 | 1.14 | 0.52 – 2.53 | 0.739 | 1.09 | 0.48 – 2.47 | 0.837 | 1.20 | 0.53 – 2.75 | 0.661 | 1.36 | 0.53–3.52 | 0.519 |
|  | 25 – 34 | 1.03 | 0.63 – 1.71 | 0.892 | 1.14 | 0.69 – 1.88 | 0.614 | 1.10 | 0.67 – 1.82 | 0.700 | 1.04 | 0.60 – 1.80 | 0.901 |
|  | 35 – 44 | 0.86 | 0.53 – 1.41 | 0.549 | 0.96 | 0.59 – 1.55 | 0.856 | 0.96 | 0.59 – 1.58 | 0.888 | 0.89 | 0.52 – 1.48 | 0.625 |
|  | 45+ | ref | --- | --- | ref | --- | --- | ref | --- | --- | ref | --- | --- |
| **Marital Status** | Single | 2.01 | 1.13 – 3.57 | 0.018 | 1.92 | 1.04 – 3.55 | 0.038 | 1.81 | 0.99 –3.31 | 0.054 | 1.47 | 0.76 – 2.81 | 0.248 |
|  | Married | ref | --- |  | ref | --- |  | ref | --- | --- | ref | --- | --- |
| **Formal Education** | No | 1.08 | 0.58 – 1.98 | 0.810 | 1.00 | 0.54 – 1.87 | 0.986 | 0.94 | 0.51 – 1.78 | 0.871 | 1.12 | 0.56 – 2.25 | 0.698 |
|  | Yes | ref | --- |  | ref | --- |  | ref | --- | --- | ref | --- | --- |
| **Employment Status** | Unemployed | 0.96 | 0.60 – 1.54 | 0.880 | 1.00 | 0.62 – 1.61 | 0.995 | 1.09 | 0.67 – 1.77 | 0.734 | 1.07 | 0.64 – 1.79 | 0.801 |
|  | Employed | ref | --- |  | ref | --- |  | ref | --- | --- | ref | --- | --- |
| **Income**  **Categories** | Lowest | 1.52 | 0.94 – 2.44 | 0.087 | 1.49 | 0.91 – 2.43 | 0.113 | 1.28 | 0.76 – 2.14 | 0.356 | 1.42 | 0.83 – 2.41  2. | 0.197 |
|  | Middle | 1.72 | 1.11 – 2.67 | 0.015 | 1.70 | 1.10 – 2.62 | 0.018 | 1.60 | 1.03 – 2.50 | 0.038 | 1.63 | 1.01 – 2.64 | 0.045 |
|  | Highest | ref | --- | --- | ref | --- | --- | ref | --- | --- | ref | --- | --- |
| **Psychological Distress**  **In the past 30 days** | High | --- | --- | --- | --- | --- | --- | 3.26 | 2.01 – 5.27 | < 0.001 | 2.29 | 1.33 – 3.95 | 0.003 |
|  | Moderate | --- | --- | --- | --- | --- | --- | 1.40 | 0.83 – 2.37 | 0.202 | 1.19 | 0.66 – 2.14 | 0.569 |
|  | Low | --- | --- | --- | --- | --- | --- | ref | --- | --- | ref | --- | --- |
| **Odd Beliefs /Magical** | Yes | --- | --- | --- | --- | --- | --- | --- | --- | --- | 5.21 | 3.32 –8.19 | <0.001 |
|  | No | --- | --- |  | --- | --- | --- | --- | --- | --- | ref | --- | --- |

Note. Odds ratio (OR) and corresponding 95% Confidence Intervals (95% CI). Reference group (ref) is the comparison group. All models are weighted.

Model 1 (n=1010) presents the adjusted ORs controlling for socio-demographics only

Model 2 (n=1010) presents the adjusted ORs controlling for socio-demographics and ethnicity

Model 3 (n=1004) presents the adjusted ORs controlling for socio-demographics, ethnicity, and psychological distress

Model 4 (n=1004) presents the adjusted ORs controlling for socio-demographics, ethnicity, psychological distress, and Odd Beliefs or Magical Thinking

^§^Lowest tertile of monthly income is <30K Qatari Riyals for non-migrants (Qataris) and <10K Qatari Riyals for migrants; Middle tertile of monthly income 30K – 50K Qatari Riyals for non-migrants (Qataris) and 10K – 20K Qatari Riyals for migrants; Highest tertile of monthly income is 50K+ Qatari Riyals for non-migrants (Qataris) and 20K+ Qatari Riyals for migrants

^¶^ Lowest tertile of psychological distress (K6) corresponds to a score range of 0 to < 8; Middle tertile of psychological distress (K6) corresponds to a score range of 8 to 9; Highest tertile of psychological distress (K6) corresponds to a score range of >9 to 24
