# Supplementary material for: Lifetime prevalence and potential determinants of psychotic experiences in the general population of Qatar
Source: Psychol Med. 2019 May 28;50(7):1110–20. doi: 10.1017/S0033291719000977 (PMC7253618; doi:10.1017/S0033291719000977)
Supplement: Supplementary file 1 [file S0033291719000977sup.zip › S0033291719000977sup002.docx]

**UNUSUAL EXPERIENCES**

**[INTERVIEWER: PLEASE READ THE FOLLOWING INTRODUCTION SLOWLY]**

The next questions are about unusual experiences, like seeing visions or hearing voices. In some cases, these experiences may be short-lived, while may reoccur over months or even years in other cases. There is limited information about the different types, causes, and frequency of occurrence of these experiences in society. Please take your time and think carefully before answering the following questions.

Importantly, please do not count times you had these experiences when you were dreaming, half-asleep, or under the influence of substances or drugs or having a fever. With these exclusions in mind, did you ever in your life have any of the following experiences?

{Q:PS1a} The first one is seeing a vision – that is, something other people who were there could not see. We do not mean having good eyesight, but rather seeing things that other people said were not there, like seeing a face, people, or strange objects. With that definition in mind, did you ever see a vision?

(INTERVIEWER KEY PHRASE: saw a vision)

1. Yes

5. No

1. Don’t Know
2. Refuse

{Q: PS1b}

The next one is hearing voices that other people could not hear. We do not mean having good hearing, but rather hearing things like strange voices coming from inside your head talking to you or about you, or voices coming out of the air when there was no one around. Did you ever have this experience?

(INTERVIEWER KEY PHRASE: heard voices)

Yes

5. No

1. Don’t Know
2. Refuse

{Q:PS1c}

Did you ever have the experience that some mysterious force was inserting strange thoughts that were definitely not your own thoughts, directly into your head?

(INTERVIEWER KEY PHRASE: inserting thoughts)

1. Yes

5. No

1. Don’t Know
2. Refuse

{Q:PS1d} Did you ever believe your thoughts were being stolen out of your mind by some strange force?

(INTERVIEWER KEY PHRASE: thoughts stolen)

1. Yes

5. No

1. Don’t Know
2. Refuse

{Q: PS1e} Did you ever feel that your mind was being taken over by strange forces that were making you do things you did not choose to do؟

(INTERVIEWER NOTE KEY PHRASE : mind taken over)

1. Yes

5. No

1. Don’t Know
2. Refuse

{Q:PS1f} Did you ever think that some strange force was trying to communicate directly with you by sending special signs or signals that you could understand but that no one else could understand. Sometimes this happens by special signs coming through the radio or television. Did you ever experience these kinds of attempts at communication from strange forces? (INTERVIEWER NOTE KEY PHRASE: experienced attempts at communication from strange forces)

1. Yes

5. No

1. Don’t Know
2. Refuse

{Q:PS1g} Did you ever believe that there was an unjust plot going on to harm you or to have people follow you that your family and friends did not believe was true?

(INTERVIEWER KEY PHRASE: plot to harm you)

1. Yes

5. No

1. Don’t Know
2. Refuse

التجارب الحسيه الإدراكية غير المعتادة

**]توجيهات للمحاور: اقرأ المقدمة التالية على مهل [**

سأسألك عن بعض التجارب الحسيه أو الإدراكية غير المعتادة مثل الرؤيا وسماع أصوات غير مألوفة .قد تستغرق هذه الحالات أيام قليلة ، ومن ثم تختفي. وفي بعض الأحيان قد تتكرر هذه الحالات على مر الشهور والسنين. لدينا القليل من المعلومات عن أنواع و كيفية حدوث هذه الحالات ومدى تكرارها في المجتمع، ولكن يضن الباحُثون بانها أكثر شائعة عن ما كان يعتقد به سابقا. الرجاء فكر بهذه العبارات التالية بتمهل قبل الإجابة عنها.

**من المهم جدا** بأن لا تحتسب الأحيان التي قد مرّرت بهذه التجارب في المنام او على وشك اليقظة من النوم. كما يرجى عدم احتساب الحالات التي قد تكون نتيجة تعاطي بعض المواد أو العقاقير أو الحمى.

**(توجيهات للمحاور: في حالة الرد بالإيجاب على الأسئلة التالية أطلب من مقدم المعلومات إعطاء تفاصيل موجزة وقم بتسجيلها)**

{Q:PS1a} التجربة الحسيه الأولى تتعلق بالرّؤية والمقصود هو رؤية أشياء أو أشخاص أو قوى معينة أخرى حولك، رغم لم يكن أحد آخر معك في ذلك الوقت (الاّن) ان يراها. لا نقصد بهذا قوت النظر وكماله ولكن المقصود رويت أشياء لا يمكن لغيرك من رويتها مثل رؤية وجه معين أو أشخاص أو أشياء غريبة. عند أخذك لهذا التعريف (المفهوم) بالاعتبار, هل سبق لك أن رأيت رؤية ؟

**]توجيهات للمحاور- العبارة المهمة : رأيت رؤية[**

1 نعم 5 لا 8 لا أعلم 9 أرفض

{Q:PS1b} التجربة الحسيه التالية تتعلق بسماع أصوات لا يمكن للأخرين بسماعها. لا نقصد بهذا قوت السمع ولكن المقصود هو سماع أصوات غريبة تصدر من ذهنك تتحدث معك, تتكلم عنك ,أو تصدر من الخلاء عندما تكون بمفردك. هل سبق لك وشهدت مثل هذه التجربة ؟

**]توجيهات للمحاور- العبارة المهمة : سماع أصوات[**

1 نعم 5 لا 8 لا أعلم 9 أرفض

{Q:PS1c} التجربة التالية تتعلق بتداخل شخص أو قوة معينة أو شيء آخر في أفكارك كإدخال افكار غريبتا عنك مباشرة بذهنك؟ هل سبق وشهدت بمثل هذه التجارب ؟

**]توجيهات للمحاور- العبارة المهمة : تداخل في أفكارك[**

1 نعم 5 لا 8 لا أعلم 9 أرفض

{Q:PS1d} التجربة التالية تتعلق بخصوصية أفكارك و القدرة على الحفاظ عليها. هل سبق وراودتك مخاوف بأن أفكارك قد تسلب من ذهنك بواسطة شخص أو شيء آخر أو قوة معينة ؟

**]توجيهات للمحاور- العبارة المهمة : أفكارك قد تسلب من ذهنك[**

1 نعم 5 لا 8 لا أعلم 9 أرفض

{Q: PS1e}هل سبق لك الشعور بأن أفكارك أو مشاعرك أو أفعالك خارجه عن إرادتك أو يتحكم فيها أشخاص أو جهات أو مخلوقات أو قوة معينة؟

**]توجيهات للمحاور- العبارة المهمة : خارجه عن إرادتك**[

1 نعم 5 لا 8 لا أعلم 9 أرفض

PS1f} {Q:هل سبق لك أن تلقطت من شخص أو قوة معينة أو شيء آخر محاولات بالتواصل معك مباشرتاً عن طريق أرسال علامات أو رموز مميزه بالنسبة لك و لا يمكن للأخرين فهمها ؟ قد تحدث هذه المحاولات بواسطة علامات أو رموز تبعث لك عن طريق التلفاز أو الهاتف. هل سبق وشهدت بمثل هذه المحاولات من قبل شخص أو قوة معينة أو شيء آخر حولك ؟

1 نعم 5 لا 8 لا أعلم 9 أرفض

{Q:PS1g}هل سبق وراودتك شكوك بأن هناك من يلاحقك أو يتجسس عليك أو يحاول إيذائك أو يخطط ضدك أو يتآمر عليك ؟

**]توجيهات للمحاور- العبارة المهمة : يتآمر عليك** [

1 نعم 5 لا 8 لا أعلم 9 أرفض
